# Linalool Induces Cell Cycle Arrest and Apoptosis in Leukemia Cells and Cervical Cancer Cells through CDKIs

**DOI:** 10.3390/ijms161226089

**Published:** 2015-11-26

**Authors:** Mei-Yin Chang, Den-En Shieh, Chung-Chi Chen, Ching-Sheng Yeh, Huei-Ping Dong

**Affiliations:** 1 Department of Medical Laboratory Science and Biotechnology, School of Medical and Health Sciences, Fooyin University, Ta-Liao District, Kaohsiung 83102, Taiwan; ft050@fy.edu.tw; 2 Department of Biotechnology, Collage of Pharmacy and Health Care, Tajen University, Yanpu Township, Pingtung County 90741, Taiwan; deshieh@tajen.edu.tw; 3 Department of Nutrition and Health Science, School of Medical and Health Sciences, Fooyin University, Ta-Liao District, Kaohsiung 83102, Taiwan; janson.yeh@msa.hinet.net; 4 Department of Physical Therapy, School of Medical and Health Sciences, Fooyin University, Ta-Liao District, Kaohsiung 83102, Taiwan; mt067@fy.edu.tw

**Keywords:** linalool, apoptosis, flow cytometry (FCM), cyclin-dependent kinases inhibitors (CDKIs), cyclin-dependent kinases (CDKs), genechip

## Abstract

*Plantaginaceae*, a popular traditional Chinese medicine, has long been used for treating various diseases from common cold to cancer. Linalool is one of the biologically active compounds that can be isolated from *Plantaginaceae.* Most of the commonly used cytotoxic anticancer drugs have been shown to induce apoptosis in susceptible tumor cells. However, the signaling pathway for apoptosis remains undefined. In this study, the cytotoxic effect of linalool on human cancer cell lines was investigated. Water-soluble tetrazolium salts (WST-1) based colorimetric cellular cytotoxicity assay, was used to test the cytotoxic ability of linalool against U937 and HeLa cells, and flow cytometry (FCM) and genechip analysis were used to investigate the possible mechanism of apoptosis. These results demonstrated that linalool exhibited a good cytotoxic effect on U937 and HeLa cells, with the IC_50_ value of 2.59 and 11.02 μM, respectively, compared with 5-FU with values of 4.86 and 12.31 μM, respectively. After treating U937 cells with linalool for 6 h, we found an increased sub-G1 peak and a dose-dependent phenomenon, whereby these cells were arrested at the G0/G1 phase. Furthermore, by using genechip analysis, we observed that linalool can promote *p53*, *p21*, *p27*, *p16*, and *p18* gene expression. Therefore, this study verified that linalool can arrest the cell cycle of U937 cells at the G0/G1 phase and can arrest the cell cycle of HeLa cells at the G2/M phase. Its mechanism facilitates the expression of the cyclin-dependent kinases inhibitors (CDKIs) *p53*, *p21*, *p27*, *p16*, and *p18*, as well as the non-expression of cyclin-dependent kinases (CDKs) activity.

## 1. Introduction

According to a statistical report by the World Health Organization, cervical cancer is the second most common cancer in women. More than 250,000 women are estimated to die annually from cervical cancer [1]. Leukemia is a serious and often lethal form of hematological malignancy [2]. In Taiwan, deaths caused by leukemia represent 2.61% of the total number of cancer mortalities, and leukemia has the highest treatment cost. Due to the promotion of cancer screenings, early cancer detection, and improvements in cancer therapy and care, the lives of cancer patients are increasingly able to be extended, which also contributes to increased odds of those patients developing other types of cancers. Cervical cancer and leukemia are both common types of second primary cancers. Thus, more effective medication is required for treating cervical cancer and leukemia [1]. The development of effective medication will inevitably become a primary focus of biotechnology, and the advancement of drug activation will enhance the functions of drugs [3,4,5].

Plants have a long history of use in the treatment of cancer, and it is significant that more than 60% of currently used anti-cancer agents come from natural sources [6,7,8]. For example, Camptothecin extracted from *Camptotheca acuminata* suppresses topoisomerase I, repressing the DNA of slow growing tumors [6,7,8]; purified etoposide (VP-16) and teniposide (VM-26) from the Mandrake [9,10] suppresses topoisomerase II and so are used in the treatment of fast-growth tumors [11,12]; Taxol extracted from *Taxus brerifolia* can stop p53-independent G2-M and cause cell apoptosis [13]; and Vinblastine and quercetin extracted from *Catharanthus roseus* also have anti-cancer applications [14,15].

Anti-cancer drugs can induce apoptosis in many tumor cells. Nowadays, researchers are still attempting to find the mechanisms responsible for drug-induced cell death. Previous studies have demonstrated the cytotoxic effect of *Plantaginaceae*, a type of traditional medicinal plant [16,17,18,19,20]. Linalool is a member of the monoterpenoids family of compounds that come from medicinal plants and are recognized as a group of potential chemopreventive compounds. Linalool is one such compound that has been reported to inhibit the growth of various human cancer cells by causing apoptotic cell death [21,22,23]. However, despite the accumulation of data, the molecular mechanism by which linalool acts against leukemia and cervical cancer cells is still unexplored. In this study, we investigate the cytotoxic effect of linalool against U937 and HeLa cells. Through flow cytometry and genechip analyses, we were able to determine how linalool suppresses the regulation of both leukemia and cervical cancer cell cycles.

## 2. Results and Discussion

### 2.1. Cytotoxicity Assay

**Figure 1:**
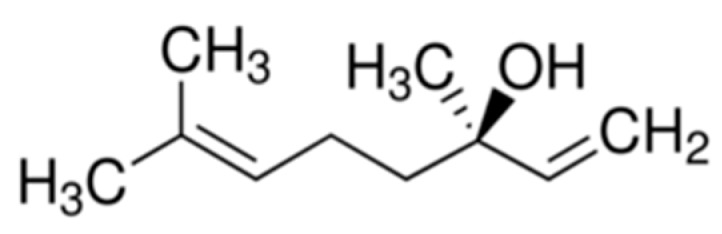
The chemical structure of linalool.

**Figure 2:**
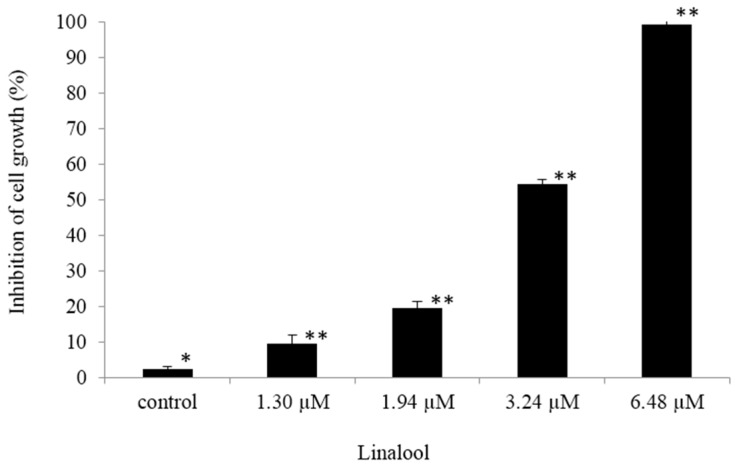
Water-soluble tetrazolium salts (WST-1) analysis was used to determine inhibition of cell growth by varying the concentrations (1.30, 1.94, 3.24, and 6.48 μM) of linalool administered to U937 cells (1.2 × 10^5^/mL) and activating for 24 h. Data are presented as mean ± SD, *n* = 3. Asterisks indicate the statistical significance between control and linalool treatment groups; * *p* ≤ 0.01 and ** *p* < 0.001 against the control.

**Figure 3:**
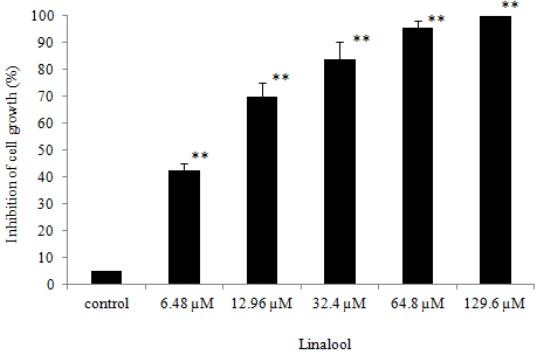
WST-1 analysis was used to determine inhibition of cell growth by varying the concentrations (6.48, 12.96, 32.4, 64.8, and 129.6 μM) of linalool administered to HeLa cells (1 × 10^5^/mL) and activating for 24 h. Data are presented as mean ± SD, *n* = 3. Asterisks indicate the statistical significance between control and linalool treatment groups; * *p* ≤ 0.01 and ** *p* < 0.001 against the control.

HeLa cell lines are transformed by Human papillomavirus 18 [24], and Human papillomavirus types 16 and 18 are the most common strains in Taiwan, accounting for nearly 70% of all cervical cancer cases [1]. Acute myeloid leukemia (AML) is the most common form of leukemia in adults; therefore, human AML cell line U937 was used as the raw material in our study. Cell viability may be observed to assess the ability of specific drugs to eliminate tumor cells. Understanding the mechanism of apoptosis can be applied in the development of anti-cancer drugs or the treatment of cancer. In our previous study, we found that normal lymphocytes can induce anticancer activity after linalool treatment [20]. Based on this theoretical foundation, linalool was investigated in this study to determine its actual mechanisms in causing the cytotoxic effect on U937 and HeLa cells. Through cell viability assay, WST-1 analysis, linalool (Figure 1) showed a good cytotoxic effect against U937 (Figure 2) and HeLa (Figure 3) cells with IC_50_ value of 2.59 and 11.02 μM, respectively, compared with 5-fluorouracil (5-FU), one of the standard therapy for cervical cancer, with value of 4.86 and 12.31 μM, respectively.

### 2.2. Cell Damage Assay

**Figure 4:**
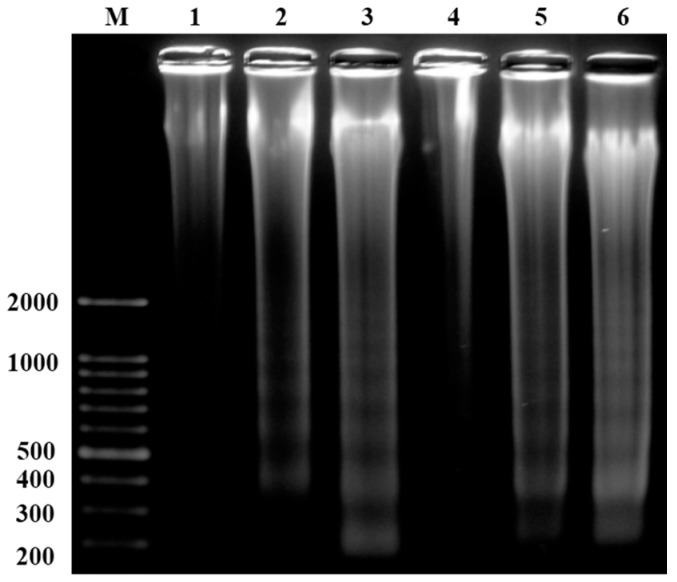
Linalool administered to HeLa cells at concentrations of 6.48 μM (**2**) and 12.96 μM (**3**) and activated for 6 h. Linalool administered to U937 cells at concentrations of 1.94 μM (**5**) and 3.24 μM (**6**) and activated for 6 h. The DNA damage following cell apoptosis was determined via agarose gel electrophoresis. Columns **1** and **4** show the control group, and **M** shows the DNA marker group.

The above results confirmed the excellent cytotoxic potential of linalool. Further investigations were conducted to determine whether linalool facilitates DNA fracturing in U937 and HeLa cells. In a DNA ladder experiment, 1.3–12.96 μM of linalool were used to process the cells for 6 h, followed by DNA extraction and electrophoresis. The typical characteristics of apoptosis, using the ladder model, were observed via the agarose gel (Figure 4). The experimental results further showed the DNA ladder model became increasingly prominent during apoptosis with an increase in dosage, allowing for clearer observation of the apoptotic characteristics in the model. This suggests that the experiment was significantly dose-dependent. Although the agarose gel electrophoresis method can be employed to observe the DNA ladder model when cells undergo apoptosis, it does have some limitations. Electrophoresis results are relatively unclear when evaluating quantity. The utility of electrophoresis for analyzing DNA is limited because only the portion of the DNA that undergoes apoptosis or decomposes can be observed. The common disadvantage of electrophoresis and cytotoxic effect analysis is that the number of cells undergoing apoptosis cannot be clearly determined; in other words, their results in terms of quantity evaluation are weaker. Because the cytotoxic effect analysis only revealed cytotoxic effects on the U937 and HeLa cells after 24 h of reaction with linalool, whether the apoptosis pathways were activated and how many cells died via the apoptosis pathways remained unknown. The best observation time for apoptosis in the electrophoresis method was 6 h after the reaction with linalool. However, the DNA available when using electrophoresis was limited; thus, only the portion of the DNA that underwent apoptosis or decomposed could be observed.

### 2.3. Cell Growth Assay

**Figure 5:**
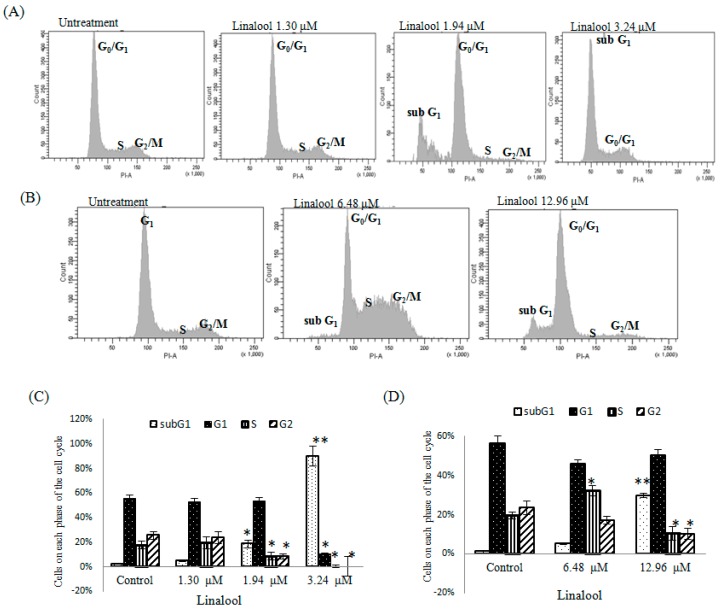
Flow cytometry (FCM) was used to obtain the apoptosis rate (sub-G1) and cell cycle distribution (G0/G1, S, and G2/M). This figure shows the profile (**A**) and percentage (**C**) of 10,000 U937 cells reacting for 6 h after adding 1.30, 1.94, and 3.24 μM concentrations of linalool and the profile (**B**) and percentage (**D**) of 10,000 HeLa cells reacting for 6 h after adding 6.48 and 12.96 μM concentrations of linalool. Data are presented as mean ± SD for the three independent experiment results. * *p* < 0.05 and ** *p* < 0.001 against control (0.1% ethylalcohol), respectively.

The FCM method is the most economical method for quantifying cell apoptosis and it can be used to analyze the amount of apoptosis. FCM results can be used in conjunction with data obtained from cell morphology for the cross-verification and confirmation of whether apoptosis is present. Therefore, the FCM method was employed to further test the linalool’s influence on the cell cycles of U937 and HeLa cells. The degree of cancer cell DNA destruction and the changes in cell cycles were observed after administering linalool in order to verify whether this substance caused U937 and HeLa cells to undergo apoptosis or suspended cell cycle. From the results shown in Figure 5, linalool administered to U937 cells in quantities of 1.30, 1.94, and 3.24 μM for 6 h, and linalool administered to HeLa cells in quantities of 6.48 and 12.96 μM for 6 h clearly presented the occurrence of sub-G1 peaks and drug dependency. The U937 cells in phase sub-G1 increased from a percentage of 4.62% to 89.88% of the overall cells. In contrast, the sub-G1 phases for the control group (DMSO processed) occupied only 1.91% of the overall cells (Figure 5A,C). The HeLa cells in phase sub-G1 increased from 5.12% to 29.51% of the overall cells, while the sub-G1 phases for the control group (DMSO processed) occupied only 1.05% of the overall cells (Figure 5B,D). Moreover, as shown in Figure 5A, where linalool was administered to U937 cells in quantities of 1.30, 1.94, and 3.24 μM for 6 h, cells in phases G0/G1 to S reduced dramatically. These phases reduced by 50% for doses of 1.30 and 1.94 μM, which suggest that a significant suppression effect occurred. However, the cell cycle distributions of cells in phase S were similar under the effects of varying quantities of linalool. Accordingly, these results imply that linalool can effectively suspend the cell cycle of U937 cells in phase G0/G1 and subsequently present sub-G1 peaks, which symbolize the occurrence of apoptosis.

### 2.4. Apoptosis Pathway Assay

**Figure 6:**
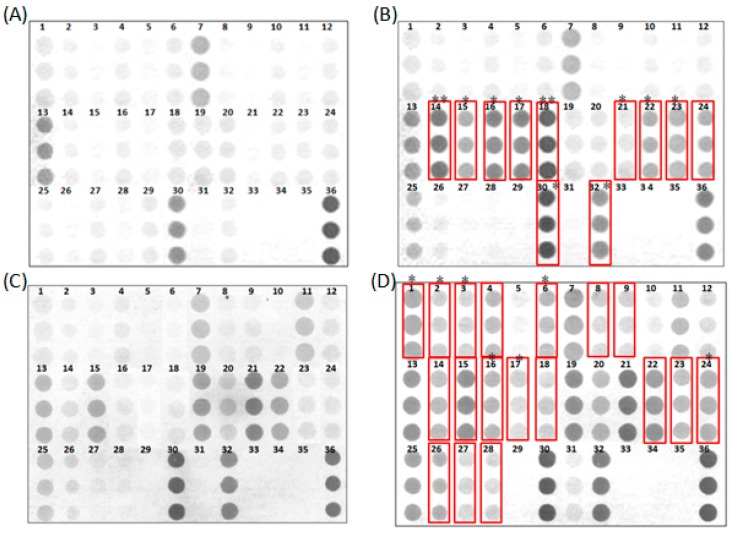
Images from the Apoptosis Pathway Detection Chip assay. U937 cells and HeLa cells were incubated with linalool at 3.24 and 12.96 μM, respectively, for 6 h, and then the mRNA expression was measured by genechip assay: (**A**) control (unstimulated) U937 cells; (**B**) linalool (3.24 μM) treated U937 cells; (**C**) control (unstimulated) HeLa cells; and (**D**) linalool (12.96 μM) treated HeLa cells. Probes 1–32: genes listed in Table 1; probes 33–34: blank; probe 35: negative control; probe 36: positive control (β-actin). Red boxes: overexpressed genes. * *p* < 0.05 and ** *p* < 0.001 against control, respectively.

Linalool was administered to HeLa cells at concentrations of 6.48 and 12.96 μM for 6 h. This induced cells in phases G0/G1 to S to reduce dramatically and present sub-G1 peaks, again indicating the occurrence of apoptosis (Figure 5). Based on these results, linalool can effectively cause U937 and HeLa cells to undergo apoptosis. To gain mechanistic insights into the linalool-mediated apoptosis of cancer cells, we used a regulated apoptosis pathway-related gene chip array to identify U937 and HeLa cells incubated with linalool at 3.24 and 12.96 μM, respectively, for 6 h. Then the mRNA expression was measured by Apoptosis Pathway Detection Chip assay (Table 1). The overexpression of *p53*, *p21*, *p27*, *p16*, *p18*, *caspase 9*, and *caspase 3* was found in both U937 and HeLa cells after treatment with linalool. However, *Bax*, *caspase 8*, *MMP-9*, and *E-cadherin* levels were increased only in U937 cells but not in HeLa cells (Figure 6; Table 1).

Different natural products, such as resveratrol, curcumin, and diallyl trisulfide, have been studied and found to induce apoptosis, accompanied by the activation of caspases in malignant cells [25,26]. Caspase plays a central role during apoptosis. In the present study, overexpression of initiator caspases 8 and 9 (*CASP8* and *CASP9*) and effector caspase 3 (*CASP3*) were also observed. The results indicated that linalool not only reduces the expression levels of *Bcl-2* and *Bcl-XL* genes but also enhances the drug‘s toxicity on U937 and HeLa cells. In addition, U937 and HeLa cells show that the overexpression of *p53*, *p21*, *p27*, *p16*, and *p18* genes causing the cell cycle to arrest at the G0/G1 and G2/M phases, respectively. Meanwhile, non-expression of the cyclin-dependent kinases (CDKs) *cyclin D*, *cyclin E*, and *cyclin A* also indirectly affects cells, causing them to stop at the G0/G1 phase. The *p21*, *p27*, *p16*, and *p18* genes have recently been discovered [27] to be important cyclin-dependent kinase inhibitors (CDKIs) and are all candidate tumor suppressor genes located downstream of *p53*. CDKIs regulate cell cycle as well as DNA replication and repair and, therefore, cannot proceed through G0/G1 if the damaged DNA is not repaired. This results in reduced replication and accumulation of damaged DNA and enhanced tumor suppression because the latter is closely related to cell cycle control. However, expression without increases in *Bax*, *caspase 8*, *MMP-9*, and *E-cadherin* in HeLa cells will be studied further in future experiments.

Regarding apoptosis, a review of the literature on linalool found in monoterpenoids showed that the majority of related studies examined the effects of *Candida albicans* [28,29,30], *Escherichia coli*, and *Staphylococcus aureus* [31,32] or the production of spices [33,34], but few studies investigated anti-cancer activity [35,36,37,38]. Our results suggest that linalool can induce the cell cycle of U937 cells to arrest at the G0/G1 phase, while HeLa cells arrest at the G2/M phase, and its function facilitates the expression of *p53*, *p21*, *p27*, *p16*, and *p18* (CDKIs) and the non-expression of CDK activity. Therefore, linalool can inhibit the cell cycle of leukemia cells and cervical cancer cells, and we believe that it could thus be used to develop novel therapeutic agents for tumors.

**Table 1:** The oligonucleotides for Apoptosis Pathway Detection Chip and the expression level of each probe.

No.	Gene Name	Oligo	Folds	
			U937	HeLa
1	*EGF*	CTGTAGGGGAAAAGGACAGTAATGACTAAGAAACTCCGAAGCCTC	<1	1.44
2	*EGFR*	AGCGGTGCTATCCTTAGGTATTCCACATTCTCAGCTGTGGGCTATTGGTC	<1	1.23
3	*Erk-1 (MAPK3)*	GAGCCAGCGCTTCCTCCACTGTGATCCGTTTATTGGGGTTAAAGGTTAAC	<1	1.22
4	*Erk-2 (MAPK1)*	CGAGGAACAGCTCACAGCCCTAACACAAGTTACCACATGCAGAGCAAATC	<1	1.24
5	*PI3K (PIK3CA)*	GCTGTTGAACTGCAGTGCACCTTTCAAGCCGCCTTTGCACTGAATTTGCA	<1	<1
6	*AKT (AKT1)*	CATCTGGGCCGTGAACTCCTCATCAAAATACCTGGTGTCAGTCTCCGACG	<1	1.32
7	*PTEN*	GTGTCATGCATGCAGATGGAAGGGGTGGAACTGTGCACTAAAGTGGGGGC	<1	1.15
8	*B-catanin (CTNNB1)*	AAACTCAGCTTGGTTAGTGTGTCAGGCACTTTCTGAGATACCAGCCCACC	<1	1.19
9	*COX-2 (PTGS2)*	ACAAACCCCGTACAGTTCTCTCTGAGGCACTAGCCTCTTTGCATCCATCT	<1	1.15
10	*Cyclin D1 (CCND1)*	CGTGCCTGGAAGTCAACGGTAGCAGCGCAATAAGAAAATGGAGCT	<1	<1
11	*Cyclin E (CCNE1)*	TTTCTTTGCTCGGGCTTTGTCCAGCAAATCCAAGCTGTCTCTGTGGGTCT	<1	<1
12	*Cyclin A (CCNA2)*	AGGTAGGTCTGGTGAAGGTCCATGAGACAAGGCTTAAGACTTTCCAGGGT	<1	<1
13	*Cyclin B*	CCGACCCAGTAGGTATTTTGGTCTGACTGCTTGCTCTTCCTCAAGTTGTC	<1	<1
14	*p21 (CDKN1A)*	GTGGCATGCCCTGTCCATAGCCTCTACTGCCACCATCTTAAAATGTCTGA	2.08	1.21
15	*p27 (PSMD9)*	GGAATTCGCCCAATAGGAAGGCTTTGGAATTGAGTGTGAGAACCTGTGGC	1.55	1.21
16	*p16 (CDKN2A)*	ACCTTCGGTGACTGATGATCTAAGTTTCCCGAGGTTTCTCAGAGC	1.80	1.23
17	*p18 (CDKN2C)*	TCTGGCCGCATCATGAATGACAGCGAAACCAGTTCGGTCTTTCAAATCGG	1.87	1.32
18	*p53*	GGCCCCTACCTAGAATGTGGCTGATTGTAAACTAACCCTTAACTG	2.45	1.19
19	*Bcl-XL (BCL2L1)*	GAGTCCTGGTCCTTGCATCTTTATCCCAAGCAGCCTGAATCCCTAGTCAA	<1	<1
20	*Bcl-2*	GCTGCACTTTGAGCCATGCTGATGTCTCTGGAATCTAAAGGTCGTACCAC	<1	<1
21	*Bax (BCL2L4)*	TGCCATAATTTATGGAGGAAAAACACAGTCCAAGGCAGCTGGGGGCCTCA	1.32	<1
22	*caspase 9 (CASP9)*	CTGGGTGCAATGGTGCACGCCTGTAGTAAGAGCTACTTGGGAGGGTCACT	1.50	1.25
23	*caspase 8 (CASP8)*	ATAGTGTTATATCTAAATAGTACCATCGGCCAGGCGCGGTGGCTC	1.54	<1
24	*caspase 3 (CASP3)*	ATCTCCCGTGAAATGTCATACTGACAGCCAGTGAGACTTGGTGCAGTGAC	1.49	1.34
25	*VEGF (VEGFA)*	ATTGAAACCTTATTTCAAAGGAATGTGTGCTGGGGAGCCAGGGGATCGGG	<1	<1
26	*VEGFR (VEGFR1)*	TGTGGGCTAGGAAACAAGGCACGGGTCCCTAAAATTAACATCTCGGTGTC	<1	1.27
27	*VEGFR (VEGFR2)*	CACTGTGCCCAGCCACCCCCTCTTCCATTTTAGAAATGATGGGTACAGTA	<1	1.19
28	*MMP-2*	ATTCTTCAGGGCTCTTTCTACAGGACAGAGGGACTAGAGCTTACT	<1	1.17
29	*MMP-3*	ACCGGCAAGATACAGATTCACGCTCAAGTTCCCTTGAGTGTGACTCGAGT	<1	<1
30	*MMP-9*	AGCCCACCTCCACTCCTCCCTTTCCTCCAGAACAGAATACCAGTT	1.85	<1
31	*MMP-13*	AATAAGTGCCAAGCACCCTCCCCAAGTATCAATAGGCACTGTGGGAAGTG	<1	<1
32	*E-cadherin (CDH1)*	CCTACCCCTCAACTAACCCCCTTTAGGGCCACATTTTCTTCTTGCTCCTA	1.72	<1
-	*Bata-actin (BA)*	AACATAATCTGAGTCATCTTCTCTCTGTTGGCCTTGGGGTTCAGGGGGGC	1.00	1.00

Folds = the mean density of each probe treated with linalool was divided by the mean density of the unstimulated probe (control).

## 3. Experimental Section

### 3.1. Cell Viability Assay

U937 (ATCC^®^ CRL-1593.2™, Hnman, pleural effusion, histiocytic lymphoma) and HeLa (ATCC^®^ CCL-2™, Human, cervix, adenocarcinoma) cells according to the growth rate were prepared into 1.2 × 10^5^/mL and 1 × 10^5^/mL samples, respectively. Then, varying concentrations of linalool (molecular weight 154.25; Sigma Chemical Co., St. Louis, MO, USA) were administered; each concentration was administered 3 times. The samples were placed in 96-well plates and cultivated in an incubator at 37 °C with 5% CO_2_ for 3 days. Subsequently, a WST-1 mixture (Roche Diagnostics GmbH, Mannheim, Germany, a colorimetric, non-radioactive assay for assessing cell viability and proliferation) was introduced into the incubator. Following 2 to 4 h reaction time, an ELISA reader (Multiskan EX, Labsystems, Stockholm, Sweden) was used to test the light absorption values, which were 450 nm. For each 96-well plate, a blank-cell control group and a standard 5-FU (5-Fluorouracil; molecular weight 130.08, Sigma Chemical Co.) control group were prepared.

### 3.2. DNA Damage Assay

U937 cell samples at various concentrations of linalool (6.48 and 12.96 μM) and HeLa cell samples at various concentrations of linalool (1.94 and 3.24 μM) were placed in plates. Subsequently, DMSO (final concentration = 0.1%) was used as the control group. The cell samples were collected at 6 h. Lysis buffer and proteinase K were administered separately to each of the cell samples after the cells had been dispersed. Each mixture was left to react overnight, after which RNase was added. The DNA was extracted using a phenol and chloroform (1:1) mixture. The product was then placed in a centrifuge at 4 °C. The DNA from the cells was then analyzed using agarose gel electrophoresis and ethidium bromide, which was used to dye and contrast the agarose gel.

### 3.3. Cell Cycle Determination

U937 cell samples at various concentrations of linalool (6.48 and 12.96 μM) and HeLa cell samples at various concentrations of linalool (1.30, 1.94 and 3.24 μM) were placed in plates. Subsequently, DMSO (final concentration = 0.1%) was used as the control group. The cells were collected after 6 h and secured with 99.9% alcohol. Then, PBS (Phosphate-buffered saline), RNase (10.0 µg/mL), and Triton (0.5%) were administered separately. The supernatant was removed from the solution following centrifuge, and PI (propidium iodide; 0.5%; Sigma Chemical Co.) dye was administered. Then, the contrast solution was again centrifuged and the subsequent supernatant removed. Finally, PBS was administered to wash and disperse the solution. The solution was then passed through a 40-µm mesh filter into a test tube [39].

### 3.4. Total RNA Extraction and First Strand cDNA Synthesis

Using a High Pure RNA Isolation Kit (Roche Diagnostics GmbH, 68298 Mannheim, Germany), total RNA was extracted from U937 and HeLa cells that had been treated with linalool for 6 h. Purified RNA was quantified by OD 260 nm using an ND-1000 spectrophotometer (NanoDrop Technologies, Wilmington, DE, USA) and quantitated by Bioanalyzer 2100 (Agilent Technologies, Santa Clara, CA, USA). First-strand cDNA was synthesized from total RNA using a Roche Diagnostics Kit (Roche Diagnostics GmbH, 68298 Mannheim, Germany) [40]. Reverse transcription was performed in a reaction mixture consisting of a 2.5 μM oligo (dT) 18-mer primer, 60 μM random hexamer primer, 1 mM deoxyribonucleotide triphosphate, 10 units of Reverse Transcriptase MMLV, and 20 units of ribonuclease inhibitor. The reaction mixtures with RNA were incubated at 55 °C for 30 min, heated to 85 °C for 5 min, and then stored at 2 to 8 °C or at −15 to −25 °C until analysis.

### 3.5. Preparation of Apoptosis Pathway Detection Chip

The procedure for the design and preparation of the genechip was carried out according to our previously described method [41,42]. Visual OMP3 (Oligonucleotide Modeling Platform, DNA Software, Ann Arbor, MI, USA) was used to design probes for target genes and β-actin (BA; housekeeping gene). The oligonucleotide sequences of 32 target genes for the Apoptosis Pathway Detection Chip are listed in Table 1. The newly synthesized oligonucleotide fragments were dissolved in distilled water to a concentration of 100 mM, and applied to a BioJet Plus 3000 nL dispensing system (BioDot Inc., Irvine, CA, USA), which sequentially blotted the target oligonucleotides; (0.05 μL per spot and 1.5 mm between spots) on a SuPerCharge nylon membrane (Schleicher and Schuell, Dassel, Germany) in triplicate, and then cross-linked to the membrane using a UV Stratalinker 1800 (Stratagene, La Jolla, CA, USA).

### 3.6. Apoptosis Pathway Detection Chip Assay

First-strand cDNA were applied for biotin labeling, and the biotin-labeled probes were then hybridized with the Apoptosis Pathway Detection Chip. The hybridized chip followed washing, blocking and color development procedures using a GeneCling^®^ Enzymatic Gene Chip Detection Kit (Carygene Co., Kaohsiung, Taiwan). The Apoptosis Pathway Detection Chips were then scanned with an Epson Perfection 1670 flat bed scanner (Seiko Epson Co., Naganoken, Japan). Subsequent quantification analysis of each spot intensity was carried out using AlphaEase^®^ FC software (Alpha Innotech Co., San Leandro, CA, USA). For each sample, the Apoptosis Pathway Detection Chip hybridization was done in triplicate to ensure the reproducibility of the results. The fold ratio of each gene was calculated as follows: spot intensity ratio = the mean intensity of target gene (*n* = 3)/the mean intensity of unstimulated control (β-actin; BA).

### 3.7. Statistical Analysis

All data were analyzed using the Statistical Package for the Social Sciences Ver 22 software (SPSS Inc., Chicago, IL, USA). The significance of the differences was analyzed by a one-way analysis of variance (ANOVA), with *p* < 0.05, *p* < 0.01 or *p* < 0.001 considered significant.

## 4. Conclusions

The results obtained in this study demonstrated that linalool produced a cytotoxic effect, by inducing the cells to undergo apoptosis, triggering cell death. The majority of studies pertaining to linalool in monoterpenoids are typically based on presenting and suppressing microorganisms and few have endeavored to research aspects of cytotoxic activation. We believe that linalool offers tremendous potential for enhancing leukemia and cervical cancer treatment and provides novel starting points for future anti-cancer research.
